# Women's Risk of Repeat Abortions Is Strongly Associated with Alcohol Consumption: A Longitudinal Analysis of a Russian National Panel Study, 1994–2009

**DOI:** 10.1371/journal.pone.0090356

**Published:** 2014-03-26

**Authors:** Katherine Keenan, Emily Grundy, Michael G. Kenward, David A. Leon

**Affiliations:** 1 Faculty of Epidemiology and Population Health, London School of Hygiene and Tropical Medicine, London, United Kingdom; 2 Department of Social Policy, The London School of Economics and Political Science, London, United Kingdom; University of Pennsylvania, United States of America

## Abstract

Abortion rates in Russia, particularly repeat abortions, are among the highest in the world, and abortion complications make a substantial contribution to the country's high maternal mortality rate. Russia also has a very high rate of hazardous alcohol use. However, the association between alcohol use and abortion in Russia remains unexplored. We investigated the longitudinal predictors of first and repeat abortion, focussing on women's alcohol use as a risk factor. Follow-up data from 2,623 women of reproductive age (16–44 years) was extracted from 14 waves of the Russian Longitudinal Monitoring Survey (RLMS), a nationally representative panel study covering the period 1994–2009. We used discrete time hazard models to estimate the probability of having a first and repeat abortion by social, demographic and health characteristics at the preceding study wave. Having a first abortion was associated with demographic factors such as age and parity, whereas repeat abortions were associated with low education and alcohol use. After adjustment for demographic and socioeconomic factors, the risk of having a repeat abortion increased significantly as women's drinking frequency increased (P<0.001), and binge drinking women were significantly more likely to have a repeat abortion than non-drinkers (OR 2.28, 95% CI 1.62–3.20). This association was not accounted for by contraceptive use or a higher risk of pregnancy. Therefore the determinants of first and repeat abortion in Russia between 1994–2009 were different. Women who had repeat abortions were distinguished by their heavier and more frequent alcohol use. The mechanism for the association is not well understood but could be explained by unmeasured personality factors, such as risk taking, or social non-conformity increasing the risk of unplanned pregnancy. Heavy or frequent drinkers constitute a particularly high risk group for repeat abortion, who could be targeted in prevention efforts.

## Introduction

Despite substantial reductions in the post-Soviet period, Russia's induced abortion rate remains the highest of all Eastern European countries [1], and is more than twice as high as in the UK [2], [3]. Moreover, in the last 20 years Russian induced abortion rates (hereafter ‘induced abortion’ is referred to simply as ‘abortion’) have declined to a much lesser extent than in neighbouring countries Ukraine and Belarus [4]. The reason for this is unclear, but could be due to high contraceptive failure rates or only modest increases in the use of modern contraception [5], which are in turn driven by poor governmental support for family planning programmes [4]. One clinic-based survey estimated that repeat abortions account for approximately 60% of all abortions sought [6], higher than the official estimate of 36% in the UK in 2011 [3].

High abortion rates in Russia contribute to high rates of maternal mortality. In 2008 Russian maternal mortality was higher than in 41 other European countries, and 4–5 times higher than in the UK [7]. According to official estimates, in 2009 10% of maternal deaths in Russia were related to abortion [8], which is approximately twice as high as countries in Western Europe [9]. In Russia repeat abortion is also linked to higher incidence of sexually transmitted infections [10] and there is a link between repeat abortion and adverse outcomes in future pregnancies [11]. Therefore, research is needed to understand the determinants of abortion in Russia, particularly repeat abortion [12].

Previous studies on risk factors for abortion in Russia have been limited by using clinic-based populations with small sample sizes [6], [13], by not analysing the risks of first and subsequent (repeat) abortions separately, and by non-consideration of behavioural risk factors such as alcohol use [14]–[16]. Alcohol use may be important given the extremely high prevalence of hazardous drinking in Russia, which seriously impacts Russian mortality [17], but is also likely to have secondary effects on patterns of family building. In general, research on alcohol and abortion is sparse. The majority of studies are concerned with investigating the hypothesis that abortion leads to an increase in mental illness and substance use. Of those looking at the reverse effect, one study using US data found links between alcohol and repeat abortion [18] and another a link between binge drinking and subsequent unintended pregnancy [19]. A recent study in Ghana found that abortion-related maternal mortality was higher in women who had consumed alcohol [20]. Other US studies have found an association between illicit substance use and abortion [21], [22]. Within Russia, small cross-sectional surveys indicate an association between alcohol use and abortion in specific populations. In a Russian survey of 87 women attending an STD clinic recent abortion was associated with hazardous drinking [23]. A study comparing Russian injecting drug users (IDUs) with non-IDUs found that risky alcohol use was associated with having had multiple sexual partners and unprotected sex [24]. However, these studies are limited by their cross-sectional nature, their small sample size and unrepresentative study populations.

We analysed incidence of abortion over the period 1994–2009 using the Russian Longitudinal Monitoring Survey, a nationally representative panel study. The aim of the analysis was to investigate the longitudinal predictors of first and repeat abortion, with a particular focus on associations with alcohol use.

## Materials and Methods

### Data

The Russian Longitudinal Monitoring Survey (RLMS) [25] is a Russian household panel survey started in the early 1990s to monitor the effect of political transition on health and wellbeing. We used data from phase 2 (1994–2009, waves 5–18) longitudinally. Full details on the RLMS design are available online (http://www.cpc.unc.edu/projects/rlms-hse). At the beginning of phase 2 (1994), a three-stage probability sample was chosen consisting of 4,718 households, of which 84.3% completed interviews (lower in the Moscow/St. Petersburg regions (60.2%)). Where possible, individual interviews were conducted with all adults in the household (97% response rate in wave 5). Households were revisited approximately annually, and attempts made to follow households and individuals who moved. The population in wave 5 (1994) compared well to the 1989 census population, in terms of distribution of household size, sex, age, and urban-rural residence. Abortion rates in the RLMS were somewhat lower than national rates, but followed the same downward trend over time.

### Sample and variables

**Figure 1:**
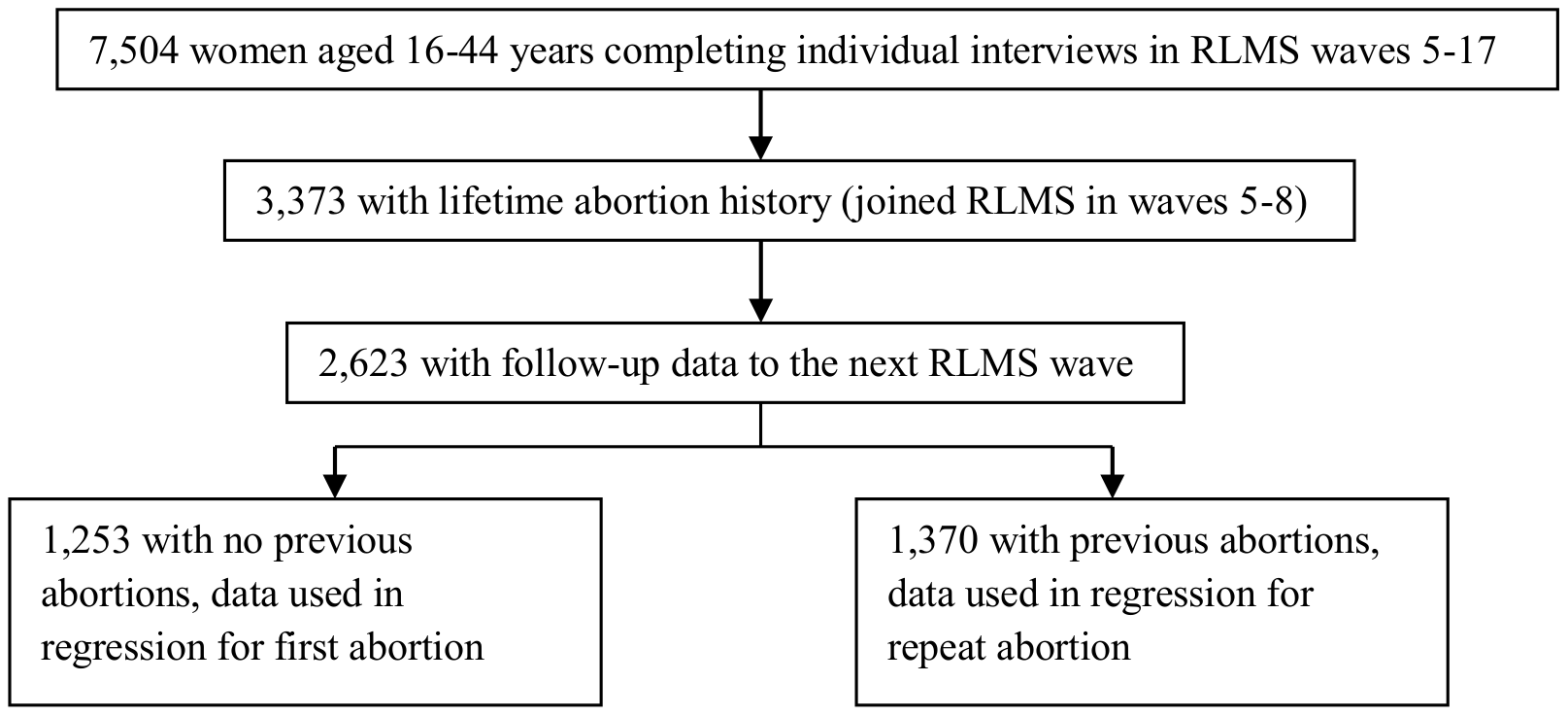
Flow diagram of sample selection.

Figure 1 shows a flow diagram for selection of women into the analysis. We excluded 4,131 women who joined the RLMS after wave 8 (1998), because data on lifetime abortion use and birth history was not collected after that point and those data were crucial in distinguishing between first and repeat abortion. Compared to those excluded, women in the analysis sample were slightly older, more likely to be married, divorced or widowed, have secondary, rather than higher education, and less likely to have abstained from alcohol in the previous 30 days. Fertility and abortion history from wave 9 was constructed longitudinally based on their earlier responses and reports of subsequent abortions or births.

The outcome events were self-reported abortion in the period between one survey wave and the following wave. At every wave, women were asked ‘*Have you had an abortion in the past 12 months?*’, and were probed to capture early gestation abortion by vacuum aspiration (known as ‘mini-abortion’ in Russia). First abortions were distinguished from repeat abortions according to whether the woman had experienced a previous abortion at the start of the follow-up period. In wave 9, questions asked about the previous 24 months, which corresponded approximately to the time since wave 8. Follow-up periods were excluded from the analysis where there was a chance that an event could be double counted (this affected 69 follow-up periods). For each follow-up period, only one abortion per woman was counted.

Alcohol use, the main factor of interest, was measured at the start of each follow-up period. Frequency of drinking was categorised into four groups: abstained/2–3 times a month/weekly/2+ times a week. We also derived a ‘drinking pattern’ variable which classified women into binge drinkers, non-binge drinkers, or abstainers. Binge drinking was defined as consuming more than 80 g of ethanol from a single type of beverage on a single occasion, a cut-off used previously in Russia [26].

Data on all covariates were taken from the start of each follow-up period. We included several variables potentially associated with drinking and with abortion: parity (no previous children, at least one child); age (5-year groups); desire for another child within the next two years (yes or no); contraceptive use [available in waves 5–12 only] (non-user, uses traditional methods (douching, calendar method or withdrawal), uses modern methods (condoms, pills, IUD, implants, diaphragm, sterilization)); education (incomplete secondary, secondary, specialist and professional, and university level and above); marital status (never married [and not cohabiting], cohabiting, married, divorced or widowed); employment (unemployed, employed or other[ which included students, housewives, etc]); household income (adjusted for household size, using the OECD-modified scale [27]), and divided into tenths); life satisfaction (not at all satisfied or less than satisfied, average or very satisfied); concerned about affording essentials in the next 12 months (very concerned versus all other categories] and smoking status (current smoker, ex-smoker or non-smoker). In the univariable analysis, we used a geographic variable which grouped Russia into four regions (central, Ural and Northwest; Moscow & St. Petersburg; Volga and the North Caucasus; and Siberia and the Far East). Variables that could be on the causal pathway between alcohol and abortion (all except age) were added individually to the multivariable models and the results carefully interpreted.

### Statistical analysis

The risk of first abortion and repeat abortion were estimated separately. We used two discrete-time hazard models [28] with robust error variance, in which the probability of first or repeat abortion between successive waves *t-1* and *t* was expressed conditionally on being at risk of the event and on alcohol use and other covariates at time *t-1*. The approach is sometimes known as ‘pooled logistic regression’. We assumed that the abortion took place anytime between consecutive waves. We tested for interactions with age and calendar time, and between alcohol and the other variables in the model. We applied separate models for each drinking variable to avoid multicollinearity.

The analysis was of complete cases, that is using only observations with available data on the previous year. As an assessment of sensitivity to the missing completely at random (MCAR) mechanism that this requires for validity, the analysis was repeated using multiple imputation for the missing data using predictors of outcome and missingness [29], which would be valid under the less restrictive Missing at Random (MAR) assumption.

### Ethics Statement

The study was approved by the London School of Hygiene and Tropical Medicine Ethics Committee (application number 6288).

## Results

The sample used for the analysis consisted of 14,229 follow-up periods, from 2,623 different women, each contributing on average 5.4 follow-up periods. Overall 475 events (abortions) were observed, 68% of which were repeat abortions. The average follow-up rate between successive waves was 83% (ranging from 71% to 96%).

Overall, 52% of women had had an abortion on entry to the RLMS (Table 1). The proportion having had an abortion increased steadily with women's age, was significantly lower among never married women, among those with no previous children, non-users of contraception, those with lower education and those in the ‘other’ employment category. Abstainers were the least likely to have had an abortion, and binge drinkers the most likely. Previous abortion use was also more common in current or ex-smokers. The same pattern of associations was seen including women without follow-up data (those with missing outcome data). Nearly half of women (46%) when surveyed had abstained from alcohol in the previous 30 days, approximately 5% had drunk more than twice a week, and approximately 14% were binge drinkers. More frequent drinking and binge drinking was significantly higher in women who were younger, cohabiting or divorced, with no children, and those who had had a previous abortion (tabulations not shown).

**Table 1:** Socio-demographic, economic and lifestyle characteristics of women according to the number of previous abortions reported on entry to the RLMS study in waves 5–8^1^.

N = 2,623	Number of previous abortions N (row %)		X^2^ test for heterogeneity P value
Characteristic	None	At least one previous abortion	
**Age (years)**			
16–19	494(96%)	18(4%)	P<0.0001
20–24	283(70%)	124(30%)	
25–29	158(44%)	204(56%)	
30–34	115(28%)	302(72%)	
35–39	118(24%)	368(76%)	
40–44	85(19%)	354(81%)	
Missing	38(84%)	7(16%)	
**Marital Status**			
Never married	589(90%)	63(10%)	P<0.0001
Cohabiting	14(40%)	21(60%)	
Married	536(33%)	1,077(67%)	
Divorced	67(30%)	158(70%)	
Widowed	9(17%)	44(83%)	
**Previous children**			
None	745(91%)	77(9%)	P<0.0001
At least one	508(28%)	1,293(72%)	
**Wants another child within the next two years**			
Yes	1,109(47%)	1,246(53%)	P = 0·0080
No	144(54%)	124(46%)	
**Current contraceptive use**			
Non-user	885(64%)	505(36%)	P<0.0001
Traditional methods^2^	102(31%)	232(69%)	
Modern methods^3^	266(30%)	633(70%)	
**Education**			
Incomplete secondary	372(54%)	321(46%)	P = 0.0001
Secondary, specialist and professional	704(46%)	823(54%)	
University and above	176(44%)	223(56%)	
Missing	1(25%)	3(75%)	
**Employment status**			
Unemployed	139(50%)	138(50%)	P<0.0001
Employed	485(33%)	992(67%)	
Other^4^	628(72%)	239(28%)	
Missing	1(50%)	1(50%)	
**Area of residence**			
Central, Ural, Northwest	535(48%)	574(52%)	P = 0.3815
Moscow & St.Petersburg	81(41%)	116(59%)	
Volga and North Caucasus	406(50%)	409(50%)	
Siberia and Far East	231(46%)	271(54%)	
**Drinking Frequency**			
Abstainer	686(58%)	507(43%)	P<0.0001
1–3 times a month	463(40%)	696(60%)	
1 occasion/week	71(37%)	119(63%)	
2+ times/week	32(43%)	42(57%)	
Missing	1(14%)	6(86%)	
**Drinking Pattern**			
Abstainer	686(58%)	507(43%)	P<0.0001
Non-binge drinker	446(42%)	613(58%)	
Binge drinker^5^	121(33%)	248(67%)	
Missing	0(0%)	2(100%)	
**Smoking**			
Current smoker	136(36%)	238(64%)	P<0.0001
Ex-smoker	99(41%)	143(59%)	
Non-smoker	1008(51%)	979(49%)	
Missing	10(50%)	10(50%)	
**Household income decile** (mean±SD)	5.3(2·8)	5.4 (2·8)	P = 0.5673
**TOTAL (row %)**	1,253(48%)	1,370 (52%)	

1 Including women with follow-up data to the next wave.

2 Traditional methods: Douching, counting days, withdrawal.

3 Modern methods: condom, oral contraceptives, IUD, implant, injectable, diaphragm, spermicide, sterilisation.

4 Includes all those who are not employed, but not seeking work, such as students, housewives, etc.

5 Reporting drinking 80 g or more of ethanol from any beverage on a single occasion.

The multivariable analyses (Table 2) show that the longitudinal predictors of first and repeat abortion were different. After adjustment for socio-demographic factors, factors related to childbearing, socio-economic factors, life satisfaction and smoking, significant risk factors for first abortion were young age, having had a previous child, and being a current smoker. Significant risk factors for repeat abortion were being aged less than 35 years, more frequent alcohol use, and low education. Abstainers were at the lowest risk of repeat abortion, and the risk increased with more frequent drinking. In similar models with drinking pattern, abstainers were at the lowest risk of repeat abortion, and binge drinkers had the highest risk (results not shown). Variables were added to the models in groups, but because adjustment did not substantially change the association with alcohol use, we present the fully adjusted models. No significant interactions were found between alcohol use and the other variables in the models. In order to explore whether the effect of alcohol was explained by contraceptive use, we restricted the analysis to data from waves 5–12, and additionally adjusted for contraceptive use (Table 3). This did not change the significance or pattern of association. After multiple imputation of missing values, the results were very similar to an analysis using complete cases (results not shown).

**Table 2:** Adjusted multivariable odds ratios for first and repeat abortion related to women's socio-demographic, economic and lifestyle characteristics at the previous wave of the RLMS study.

	**First abortion at time t Total N = 5,345**		**Repeat abortion at time t Total N = 7,843**	
**Variables at time t-1**	OR, mutually adjusted (95% CI)	**P value**	**OR, mutually adjusted (95% CI)**	**P value**
**Drinking frequency**				
Abstainer	1.00 [ref]	-	1.00 [ref]	-
1–3 times a month	0.83 (0.57–1.23)	0.3711	1.61 (1.21–2.14)	0.0008
Once/week	1.14 (0.64–2.02)	0.6546	2.16 (1.47–3.18)	<0.0001
2+ times/week	0.49 (0.17–1.42)	0.1919	2.98 (1.70–5.23)	0.0001
Test for trend	P = 0.6259		P<0.0001	
**Age (years)**				
16–19	1.00 [ref]	-	1.00 [ref]	-
20–24	0.79 (0.42–1.46)	0.4474	0.67 (0.26–1.72)	0.3903
25–29	0.57 (0.28–1.17)	0.1251	0.49 (0.18–1.35)	0.1675
30–34	0.33 (0.14–0.75)	0.0081	0.39 (0.14–1.06)	0.0661
35–39	0.25 (0.10–0.62)	0.0027	0.23 (0.08–0.65)	0.0051
40–44	0.09 (0.03–0.28)	<0.0001	0.06 (0.02–0.17)	<0.0001
**Marital Status**				
Never married	0.67 (0.35–1.26)	0.2154	0.89 (0.47–1.66)	0.7104
Cohabiting	0.91 (0.39–2.11)	0.8215	0.75 (0.42–1.34)	0.3284
Married	1.00 [ref]	-	1.00 [ref]	-
Divorced	0.99 (0.53–1.86)	0.9854	1.16 (0.78–1.73)	0.4772
Widowed	1.36 (0.31–5.98)	0.6816	0.20 (0.03–1.21)	0.0793
**Has at least one child**	3.63 (1.94–6.81)	<0.0001	1.30 (0.74–2.26)	0.3576
**Would like another child within next 2 years**	1.51 (0.93–2.46)	0.0962	0.93 (0.60–1.46)	0.7575
**Education**				
Incomplete secondary	1.00 [ref]	-	1.00 [ref]	-
Secondary, specialist and professional	1.15 (0.79–1.68)	0.4687	0.79 (0.59–1.05)	0.1050
**Employment status**				
Unemployed	1.00 [ref]	-	1.00 [ref]	-
Employed	1.15 (0.79–1.68)	0.6301	0.87 (0.58–1.33)	0.5166
Other^1^	0.69 (0.40–1.20)	0.8389	1.39 (0.87–2.20)	0.1596
**Household income decile** (continuous)	1.02 (0.96–1.09)	0.4492	1.01 (0.97–1.06)	0.1689
**Concerned about affording essentials**	0.82 (0.58–1.18)	0.2903	0.83 (0.63–1.08)	0.1690
**Poor life satisfaction**	1.00 (0.70–1.42)	0.9979	1.16 (0.89–1.52)	0.2730
**Smoking**				
Current smoker	1.00 [ref]	-	1.00 [ref]	-
Ex-smoker	0.76 (0.41–1.42)	0.3873	1.25 (0.84–1.86)	0.2732
Non-smoker	0.59 (0.37–0.93)	0.0229	0.97 (0.69–1.36)	0.8531

1 Those not in employment but not seeking work, including students, housewives, etc.

**Table 3:** Adjusted odds ratios for repeat abortion associated with women's alcohol use in the RLMS data waves 5–12, additionally adjusted for contraceptive use.

Alcohol variables	Adjusted OR (95% CI)^1^	P value
**Drinking Frequency** N = 6,158		
Abstainer	1.00 [ref]	-
1–3 times a month	1.66 (1.23–2.22)	0.0007
Once/week	2.09 (1.36–3.21)	0.0006
2+ times/week	2.83 (1.51–5.28)	0.0011
Test for trend	P<0·0001	
**Drinking Pattern** N = 6,158		
Abstainer	1.00 [ref]	-
Non-binge drinker	1.56 (1.13–2.14)	0.0061
Binge drinker	2.28 (1.62–3.20)	<0.0001
Test for trend	P<0·0001	

1 Adjusted for age, calendar time, marital status, parity, desire for more children, contraceptive use, education, employment status, household income, concern about affording essentials, life satisfaction, smoking.

**Figure 2:**
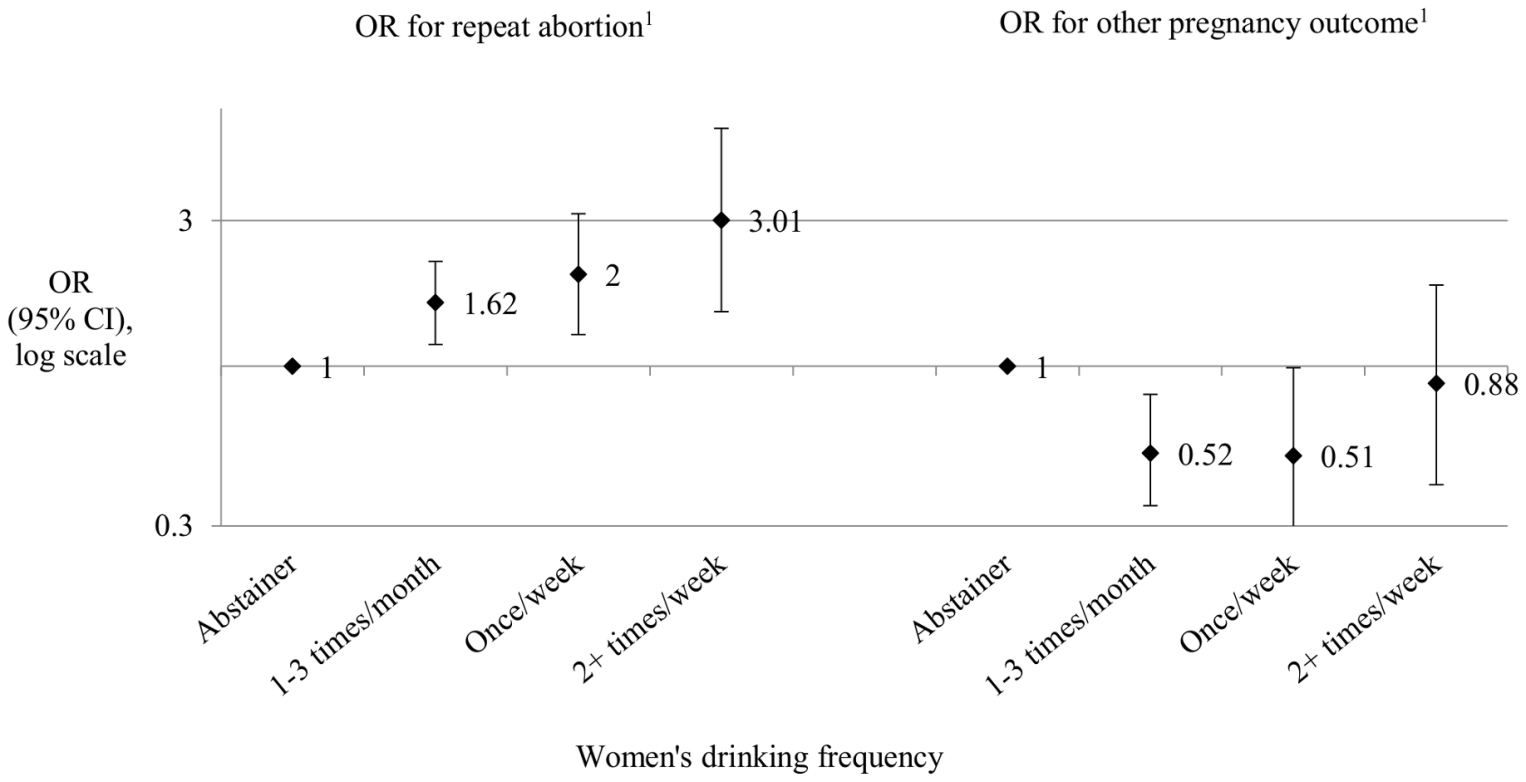
Adjusted odds ratios for repeat abortion and other type of pregnancy outcome related to drinking in the RLMS waves 5–12. ^1^Adjusted for age, calendar time, marital status, parity, desire for more children, contraceptive use, education, employment status, household income, concern about affording essentials, life satisfaction and smoking.

Because the RLMS data did not include precise dates of pregnancy or abortion, it was not possible to tell if the association was explained by higher pregnancy rates in drinkers, or by increased likelihood that drinkers will choose an abortion rather than progress with the pregnancy. To explore this we compared the association between alcohol and repeat abortion with the association between alcohol and any other type of pregnancy outcome (live births, still births and miscarriages, but not abortions) (Figure 2). If the association between alcohol and abortion was entirely explained by higher pregnancy rates, frequent drinkers in both groups would be expected to have higher rates of both types of pregnancy events. Comparison showed that this was not the case: there was an association with alcohol only in the repeat abortion group, and no association between alcohol and other pregnancy outcomes. This suggests that the association between alcohol use and repeat abortion is unlikely to be explained by increased chance of pregnancy alone, but that drinkers are more likely to choose an abortion than non-drinkers.

## Discussion

Our findings show that the determinants of first and repeat abortion in Russia over the period 1994–2009 were different. Independent of other factors including contraceptive use, having previous children significantly predicted a first abortion, suggesting that first abortions might be commonly used to space births or limit family size. However for repeat abortions these demographic factors were unimportant and instead, women's alcohol use and low education were significant independent predictors. The risk of repeat abortion showed a dose-response effect with women's drinking frequency, and the risks were elevated even for moderate drinkers.

Very few studies anywhere have explored longitudinally the association between alcohol and abortion, and of these the vast majority are concerned with investigating the hypothesis that abortion leads to an increase in substance use, rather than the other way around (for example, [30]). This study confirms that the association between alcohol and abortion found in a small sample of Russian STD clinic attendees [23] also applies at population level, but only for repeat abortion. Our findings were also consistent with previous Russian studies showing that abortion is more common in women aged less than 35 years, among those with previous children and those with low education [6], [13]–[16]. The association between alcohol and repeat abortion was not explained by higher pregnancy rates or by lower contraceptive use rates in drinkers, which was surprising given the large body of literature on the association of alcohol with unprotected sex [31], an association also found in Russian studies [23], [32].

An alternative explanation for the association between alcohol and repeat abortion could be that the experience of having a first abortion leads to an increase in alcohol use. A systematic review has found weak evidence for such an effect [33]. Moreover, the likelihood of abortion leading to increased alcohol use and psychological problems may be lower in Russia, given that abortion is relatively socially acceptable. Nevertheless, we explored this possibility in the RLMS using a subsample of women with continuous follow-up data from waves 6–11 (1995–2003), who had had no previous abortions at wave 6 (N = 337). We estimated how these women's drinking at wave 6 (1995) predicted the chance of them having had at least two abortions (i.e. becoming repeat abortion clients) by wave 11 (2003). The results showed the same pattern of effect for repeat abortion as seen in Table 2: a dose-response relationship with drinking frequency. This suggests that reverse causality is unlikely to explain the association between alcohol use and repeat abortion.

Studies from outside of Russia have suggested that the association between substance use and abortion may be explained by personality factors such as unconventionality, rebelliousness, low parental bonding, and risk-taking, [22] all of which increase the likelihood of both and thus results in an association. Similar arguments apply to the association between alcohol use and sexual risk taking [34]. This could be the case in Russia where heavy drinking is considered socially acceptable for men, but not women [35]. Female drinking could be an indicator of personality factors like unconventionality, risk taking and sensation seeking. The association between abortion use and smoking also suggests that abortion users are less health-conscious overall. To explore these hypotheses, the analysis could be repeated including factors unmeasured in the RLMS such as personality factors, mental health and family background.

This study is one of a very few that have investigated alcohol use and abortion in any population. Moreover, it is the first to explore the issue in Russia using a general population sample. The study was unique in using longitudinal data covering several years, and in exploring first and repeat abortions separately.

The study had some limitations. Contraceptive use was measured by a self-report of the method used most often in the previous 30 days, and it is possible that event-based reporting would capture non-use more effectively. Alcohol use may have been underreported. We assumed that the report of alcohol use within the previous 30 days was broadly representative of a woman's overall level of drinking. Selection bias may have occurred through differential loss to follow-up, which was higher in the early waves of the RLMS, among women aged under 25 years, those never married, those from Moscow or St. Petersburg, and those with no previous births or abortions. In addition, we used a subsample of women who were slightly older, less educated and drank less than those excluded. However, the main exposure of alcohol use was not associated with loss to follow-up, and the multiple imputation analysis suggested that the missing data did not bias the associations found in the multivariable models.

## Conclusions

We found that alcohol use in Russian women increased the likelihood of subsequently experiencing a repeat abortion, but not a first abortion. There was a dose-response effect between volume and frequency of alcohol consumed and subsequent risk of repeat abortion, independent of demographic and socioeconomic factors and contraceptive use. Given that first abortion is independently associated with having had a child, we suggest that first abortions are routinely used to space or limit births, and women that go on to have repeat abortions are distinguished by lifestyle factors that are associated with increased risk of unplanned pregnancy. Therefore alcohol use could potentially be used as a screening tool to identify women at increased risk of repeat abortion and target prevention measures most effectively. Given that Russia has one of the world's highest rates of hazardous drinking and abortion use, the association between alcohol use and repeat abortion deserves further exploration to understand the mechanism.
